# Haptic Nudges Increase Affected Upper Limb Movement During Inpatient Stroke Rehabilitation: Multiple-Period Randomized Crossover Study

**DOI:** 10.2196/17036

**Published:** 2020-07-29

**Authors:** Nada Elizabeth June Signal, Ruth McLaren, Usman Rashid, Alain Vandal, Marcus King, Faisal Almesfer, Jeanette Henderson, Denise Taylor

**Affiliations:** 1 Health and Rehabilitation Research Institute Auckland University of Technology Auckland New Zealand; 2 Department of Statistics University of Auckland Auckland New Zealand; 3 Callaghan Innovation Christchurch New Zealand; 4 Exsurgo Rehabilitation Auckland New Zealand; 5 Assessment, Treatment and Rehabilitation Department Waitakere Hospital Waitemata District Health Board Auckland New Zealand

**Keywords:** stroke, rehabilitation, physical activity, movement, disability, technology, upper limb, wearable, haptic, nudge

## Abstract

**Background:**

As many as 80% of stroke survivors experience upper limb (UL) disability. The strong relationships between disability, lost productivity, and ongoing health care costs mean reducing disability after stroke is critical at both individual and society levels. Unfortunately, the amount of UL-focused rehabilitation received by people with stroke is extremely low. Activity monitoring and promotion using wearable devices offer a potential technology-based solution to address this gap. Commonly, wearable devices are used to deliver a haptic nudge to the wearer with the aim of promoting a particular behavior. However, little is known about the effectiveness of haptic nudging in promoting behaviors in patient populations.

**Objective:**

This study aimed to estimate the effect of haptic nudging delivered via a wrist-worn wearable device on UL movement in people with UL disability following stroke undertaking inpatient rehabilitation.

**Methods:**

A multiple-period randomized crossover design was used to measure the association of UL movement with the occurrence of haptic nudge reminders to move the affected UL in 20 people with stroke undertaking inpatient rehabilitation. UL movement was observed and classified using movement taxonomy across 72 one-minute observation periods from 7:00 AM to 7:00 PM on a single weekday. On 36 occasions, a haptic nudge to move the affected UL was provided just before the observation period. On the other 36 occasions, no haptic nudge was given. The timing of the haptic nudge was randomized across the observation period for each participant. Statistical analysis was performed using mixed logistic regression. The effect of a haptic nudge was evaluated from the intention-to-treat dataset as the ratio of the odds of affected UL movement during the observation period following a “Planned Nudge” to the odds of affected limb movement during the observation period following “No Nudge.”

**Results:**

The primary intention-to-treat analysis showed the odds ratio (OR) of affected UL movement following a haptic nudge was 1.44 (95% CI 1.28-1.63, *P*<.001). The secondary analysis revealed an increased odds of affected UL movement following a Planned Nudge was predominantly due to increased odds of spontaneous affected UL movement (OR 2.03, 95% CI 1.65-2.51, *P*<.001) rather than affected UL movement in conjunction with unaffected UL movement (OR 1.13, 95% CI 0.99-1.29, *P*=.07).

**Conclusions:**

Haptic nudging delivered via a wrist-worn wearable device increases affected UL movement in people with UL disability following stroke undertaking inpatient rehabilitation. The promoted movement appears to be specific to the instructions given.

**Trial Registration:**

Australia New Zealand Clinical Trials Registry 12616000654459; https://www.anzctr.org.au/Trial/Registration/TrialReview.aspx?id=370687&isReview=true

## Introduction

Although the incidence of stroke has reduced, its burden continues to grow as more people are surviving after stroke and living with disability [1]. Direct and indirect health care costs following stroke are strongly correlated with stroke disability, with greater disability associated with greater costs [2,3]. Around 80% of stroke survivors experience upper limb (UL) disability, with only 5%-20% achieving full recovery of UL function [4-6]. UL disability has subsequent impacts on independence in activities of daily living, discharge destination, return to work, quality of life, and mood [7-10].

Effective rehabilitation involves high-dose, intensive, task-specific activity [11]. Meta-analyses of randomized controlled trials suggest there is a dose-response relationship, with higher doses of rehabilitation resulting in better outcomes [12-14]. However, studies describing usual stroke care illustrate that the dose of UL rehabilitation received by people with stroke is extremely low, with as little as 4-6 minutes in physiotherapy sessions and 11-17 minutes in occupational therapy sessions [15]. Movement of the affected UL outside formal therapy sessions during inpatient rehabilitation is also low [16]. Consequently, affected UL movement dose, both within formal therapy sessions and across the rehabilitation day, is currently insufficient to reduce UL disability following stroke.

Rehabilitation technologies have been proposed as potential solutions to the limited dose of rehabilitation this population receives [17]. A number of technological solutions have been developed for use in UL stroke rehabilitation, including virtual reality, gaming, and robotics [17-19]. Despite indications of effectiveness [20,21], the use of rehabilitation technologies is not yet pervasive in stroke care [18]. Therapists have identified several barriers to adopting rehabilitation technologies, including concerns about patient safety, whether the technology effectively addresses a clinical need, and the feasibility of technologies from time, space, and cost perspectives [22,23]. The poor uptake of rehabilitation technologies is inconsistent with research involving people with stroke that indicates rehabilitation technologies can support engagement and interest in performing repetitive rehabilitation activities and offer a means of social support [19,24,25]. Activity monitoring and promotion using wearable devices is a potential low-cost and feasible rehabilitation technology. Research investigating the effect of wearable devices on outcomes following stroke is in its infancy. Preliminary indications suggest that wearable devices may increase the amount and intensity of physical activity undertaken during rehabilitation [26-28] and potentially contribute to improved functional outcomes [29]. However, the effect of wearable devices on UL rehabilitation and outcomes has been less well studied [30,31], with much of the research to date focusing on the accuracy and validity of accelerometry measurement of real-world UL movement [32-34].

Wearable devices that deliver haptic nudges have been used to promote physical activity in both healthy and patient populations [35-39]. Commonly, a haptic nudge reminder is delivered via a small motor embedded inside a wearable device. Wearers are encouraged to respond to a haptic reminder by performing a particular behavior. For example, a haptic nudge might be used to remind the wearer to stand up and move after an extended period of sitting or to undertake rehabilitation exercises. However, despite the pervasiveness of haptic nudging in consumer wearable devices, there remains much to learn about the effectiveness of haptic nudging in promoting behaviors in patient populations. Haptic nudges have been used to effectively promote behaviors in people with autism spectrum disorder [40] and traumatic brain injury engaged in a rehabilitation task [41]. Research also suggests that wearable devices are feasible and well tolerated in people with stroke [30,42,43], with preliminary data indicating haptic nudging via wearable devices may promote affected UL movement [31]. The aim of this study was to estimate the effect of haptic nudging delivered via a wrist-worn wearable device on UL movement in people with UL disability following stroke undertaking inpatient rehabilitation.

## Methods

### Study Design

A multiple-period randomized crossover design was used to measure the association of UL movement with the occurrence of haptic nudge reminders to move the affected UL in 20 people with stroke undertaking inpatient rehabilitation. UL movement was observed and classified using movement taxonomy across 72 one-minute observation periods from 7:00 am to 7:00 pm on a single weekday. On 36 occasions, a haptic nudge (Nudge) to move the affected UL was provided just before the observation period, and on the other 36 occasions, no haptic nudge was given (No Nudge). The timing of the haptic nudge was randomized. Approval for this study was obtained from the New Zealand Health and Disability Ethics Committee (16/NTA/74).

### Participants

All people with stroke admitted to the rehabilitation service from July 2018 through December 2018 were considered for inclusion in this study. Participants were included if they had a confirmed diagnosis of stroke based on the Oxford classification system [44], presented with UL deficit as a result of stroke as determined by their rehabilitation therapist, were deemed medically stable and fit for rehabilitation by their medical consultant, and provided written informed consent. Participants were excluded if they had cognitive, behavioral, or communication impairments that, in the opinion of the research team (RM, DT, NS), would limit their ability to participate in the research (for example, if the person was unable to follow a 2-step verbal command or recall the details of the research study); were within 3 days of planned discharge from inpatient rehabilitation; or reported shoulder pain.

### Procedure

Potential participants were identified and referred to the research team by their rehabilitation therapist. They were then informed about the study and screened against the inclusion and exclusion criteria by a trained research assistant. Eligible participants provided written informed consent and identified a mutually agreeable day for data collection. Demographic, clinical, and medical information was gathered from the medical record of consenting participants by the rehabilitation therapist. Collected data included age, sex, ethnicity, date of stroke, type of stroke, side of body most affected, dominant hand prior to stroke, date of admission to the rehabilitation ward, estimated date of discharge, comorbidities, and medications.

On the day of data collection, the participant was fitted with a BuzzNudge wearable device on the wrist of the affected UL. The BuzzNudge is a Bluetooth-enabled wearable device with a 2.3 V coin vibration motor (Precision Microdrives Ltd, Model 310-103), which provided 3 consecutive vibratory stimuli of 0.3 seconds duration at 150 Hz within 1.5 seconds, representing a similar magnitude of stimulus to the vibration of a phone. The researcher explained the value of moving the affected UL after stroke and instructed the participant to “move, try and move, or visualize moving their (affected) arm” following a nudge. The researcher emphasized that the participant should do whatever movement they felt they could manage. If sensation was impaired in the affected UL such that the participant could not feel the haptic nudge, the device was worn on the less affected UL, but the participant was still instructed to use the haptic nudge as a reminder to move the affected UL.

During data collection, participants were followed discreetly out of their field of view around the rehabilitation ward, therapy areas, and hospital facilities by a trained researcher (where feasible). The researcher manually recorded UL movement for 1 minute every 10 minutes [45]. Each minute of observation was broken into 6 epochs of 10 seconds using a silent interval timer. UL movement was classified according to a previously defined taxonomy: (1) unilateral affected UL movement; (2) unilateral unaffected UL movement; (3) bimanual movement, where movement of both ULs was observed to achieve a common task or purpose; (4) bilateral limb movement, where movement of both ULs was observed to achieve independent or unrelated tasks; and (5) no movement [16]. When patients were not able to be directly observed (ie, because curtains were drawn or when in showers or toilets), activity was recorded after conferring with the participant, staff, or family members, as appropriate. In circumstances where the activity could not be estimated (eg, during 4 randomly scheduled observer breaks), activity was coded as unobserved [16,46].

Haptic nudge reminders were triggered by the researcher via Bluetooth immediately before movement observation according to a planned randomization schedule. For half of the observation periods, a haptic nudge was to be provided, and for half, a haptic nudge was not to be provided. Further details regarding the randomization schedule are presented in Multimedia Appendix 1. Haptic nudges were not triggered if the participant was not visible, the participant was asleep, or a nudge was considered inappropriate (eg, if the participant was drinking a hot beverage or undertaking an assessment procedure). Any scheduled nudge that was not given was recorded as a “Missed Nudge.”

### Statistical Analysis

Coded data were entered into a Microsoft Excel spreadsheet. Descriptive analysis was used to examine the amount and type of UL movement. If a participant withdrew from the study, their movement observations were coded as missing values, and their scheduled nudges were coded as Missed Nudges. Statistical analysis was performed using mixed logistic regression. Total affected UL movement was collated based on all observations in which the affected UL was moved: total affected UL movement = unilateral affected UL movement + bilateral limb movement + bimanual movement.

In the primary analysis using the intention-to-treat dataset, nudges were represented as a fixed effect factor with two levels: Planned Nudge (ie, Nudge + Missed Nudge) and No Nudge, meaning the analysis considered whether a nudge was planned or not, rather than delivered. The effect of Planned Nudges was evaluated as the ratio of the odds of affected UL movement during the observation period following a Planned Nudge to the odds of affected UL movement during the observation period following No Nudge. More formally, the primary null hypothesis tested with the model was: H_0_: OR_Planned Nudge/No Nudge_ = 1.

The sensitivity of the effect of the Planned Nudge to missing values was tested with the pooled effect of 10 worst-case random imputations at the level of the participant with the worst outcomes.

In the secondary analysis using the intention-to-treat dataset, two additional models were used to evaluate the effect of a Planned Nudge on unilateral affected UL movement and the sum of bilateral limb movement + bimanual movement, respectively. A post-hoc exploratory analysis with an instrumental variable approach was used to evaluate the local average treatment effect (also known as complier average causal effect) of the haptic nudge reminder. This analysis considered the effect of the haptic nudge when delivered (Nudged) compared with no haptic nudge (Not Nudged) irrespective of the schedule on total affected UL movement. To explain the variation in UL movement across the day, all models fitted the data with smooth natural splines that had 1 degree of freedom per hour. To account for correlated repeated measures, the models included hierarchical random effects per participant and per hour within participant. Statistical analyses were performed using R version 3.5.1 (R Foundation for Statistical Computing, Vienna, Austria) with *lme4* version 1.1-21 [47], *splines* version 3.5.1 [48], and *emmeans* version 1.3.4 [39]. The threshold of statistical significance was set at .05. A detailed statistical analysis report containing code snippets and additional graphical representations of data is available in Multimedia Appendix 1.

## Results

### Participant Characteristics

In total, 20 people consented to participate in this study (Table 1). Participants’ median age was 76 years (IQR 68-83 years), and the median time since stroke was 23.5 days (IQR 8.25-38.25 years); 9 participants had left hemiparesis, 10 had right hemiparesis, and 1 participant had bilateral symptoms with the left UL more affected than the right. Five participants had total anterior circulation syndrome, 10 had partial anterior circulation syndrome, 4 had lacunar circulation syndrome, and 1 had posterior circulation syndrome. Of the participating patients, 4 had hemorrhagic stroke, and 16 had ischemic stroke. Participant 2 was withdrawn from the study when 6 nudges in a row were unable to be delivered due to a technical error. Participant 6 asked to withdraw 20 minutes into data collection due to experiencing anxiety associated with wearing the device.

**Table 1:** Participant demographics.

Participant	Age range (years)	Gender	Stroke classification	Days since stroke	AUL^a^	AUL = dominant UL^b^	Device worn on AUL
1	70-79	Male	LACS-I^c^	9	Left	No	Yes
2^d^	80-89	Female	TACS-I^e^	39	Left	No	Yes
3	70-79	Female	TACS-I	59	Left	No	Yes
4	40-49	Female	LACS-H^f^	8	Right	Yes	Yes
5	60-69	Female	PACS-I^g^	5	Right	Yes	Yes
6^d^	80-89	Male	PACS-I	34	Left	No	Yes
7	70-79	Male	TACS-I	27	Right	Yes	Yes
8	80-89	Female	PACS-I	7	Left	No	Yes
9	80-89	Male	TACS-I	67	Right	Yes	Yes
10	60-69	Male	PACS-I	36	Right	Yes	Yes
11	50-59	Female	TACS-I	25	Left	Yes	Yes
12	70-79	Male	LACS-H	33	Left	No	No
13	80-89	Male	PACS-I	12	Left	No	Yes
14	60-69	Male	LACS-I	3	Right	Yes	No
15	70-79	Male	PACS-H^h^	40	Right	Yes	Yes
16	80-89	Female	PACS-I	22	Right	Yes	Yes
17	60-69	Female	PACS-I	6	Left	No	Yes
18	60-69	Male	POCS-I^i^	10	Bilateral	Yes	Yes
19	80-89	Male	PACS-I	9	Right	Yes	Yes
20	80-89	Male	PACS-H	160	Right	Yes	Yes

^a^AUL: affected upper limb.

^b^UL: upper limb.

^c^LACS-I: lacunar circulation syndrome ischemic.

^d^Participant withdrawn.

^e^TACS-I: total anterior circulation syndrome ischemic.

^f^LACS-H: lacunar circulation syndrome hemorrhagic.

^g^PACS-I: partial anterior circulation syndrome ischemic.

^h^PACS-H: partial anterior circulation syndrome hemorrhagic.

^i^POCS-I: posterior circulation syndrome ischemic.

### Data Completeness

In total, 7517 of a possible 8640 observations of movement in 10-second time intervals were recorded across the 20 participants (median 414 observations/participant, IQR 402-420 observations/participant, range 12-432 observations/participant), representing data completeness of 87.0%. Data loss was due to the 2 participants who withdrew (769/8640 observations, 8.9%) and the remaining participants being away from the service for appointments or involved in private personal hygiene activities (354/8640 observations, 4.1%).

Of the 720 Planned Nudges, 32.6% (235 Nudges) were not delivered (Missed Nudge), with 8.7% (63/720 Nudges) ascribed to the 2 participants that withdrew. For the remaining Missed Nudges, participants were sleeping for 12.6% (90/720) of the Planned Nudges; Nudges could not be directly observed for 6.7% (48/720) of the Planned Nudges; a nudge was deemed inappropriate for 2.8% (20/720) of the Planned Nudges; a technical error prevented nudging for 1.4% (10/720) of the Planned Nudges; and the reason was not stated for 0.4% (3/720) of the Planned Nudges.

### UL Movement

During observations without a nudge scheduled (No Nudge), the affected UL moved 19.2% of the time; 15.6% of movement occurred in conjunction with the unaffected UL, and only 3.6% of the time the movement was of the affected UL by itself. The unaffected UL moved 39.2% of the time, with half of this movement (23.6%) being movement of the unaffected UL by itself. Participants used one or both ULs for 42.8% of the observation time.

### Haptic Nudge Effect

The results of the statistical analyses are presented in Table 2. The treatment effect of the intervention is represented by the odds ratio (OR) for Planned Nudge versus No Nudge. This OR indicated that the odds of moving the affected UL either independently (unilateral affected UL movement) or in concert with the unaffected limb (bimanual movement or bilateral limb movement) was 1.44 times greater following a Planned Nudge than following No Nudge. The proportions estimated by the model for the affected UL movement (unilateral affected UL movement + bilateral limb movement + bimanual movement) recorded during the observation periods following a Planned Nudge and No Nudge were 26.7% (95% CI 15.4%-42.2%) and 20.2% (95% CI 11.2%-33.6%), respectively. Therefore, the average absolute increase in the proportion of affected UL movement with the intervention was 6.5% (95% CI 4.2%-8.6%), representing an increase of 32.2% in average activity. The sensitivity analysis showed that the effect of the Planned Nudge on unilateral affected UL movement + bilateral limb movement + bimanual movement was robust to missing values (*P*<.001). The proportion of observation periods with affected UL movement following Planned Nudges and No Nudges by participants is represented in Table 3.

The secondary analysis revealed that the odds of moving the affected UL independently (unilateral affected UL movement) was 2.03 times greater following a Planned Nudge than following No Nudge. However, the OR for either bilateral or bimanual movement (bilateral limb movement + bimanual movement) was only 1.13 times greater. The exploratory analysis revealed that the OR for the effect of the haptic nudge when delivered (Nudged) compared with no haptic nudge (Not Nudged) irrespective of the schedule was 1.64.

**Table 2:** Odds of an affected upper limb (UL) movement recorded during the observation periods.

Estimate		Odds ratio	95% CI	Standard error	Z value	*P* value
**Planned nudge/no nudge**						
	Primary analysis: AU^a^+BiL^b^+BiM^c^	1.44	1.28-1.63	0.09	5.86	<.001
	Sensitivity analysis: AU+BiL+BiM	1.30	1.16-1.46	0.08	4.4	<.001
	Secondary analysis: AU	2.03	1.65-2.51	0.22	6.65	<.001
	Secondary analysis: BiL+BiM	1.13	0.99-1.29	0.08	1.80	.07
**Nudged/not nudged**						
	Exploratory analysis: AU+BiL+BiM	1.64	1.42-1.89	0.12	6.77	<.001

^a^AU: unilateral affected upper limb movement.

^b^BiL: bilateral limb movement.

^c^BiM: bimanual movement.

**Table 3:** Proportion of observations with affected upper limb movment (unilateral affected upper limb movement + bilateral limb movement + bimanual movement) following No Nudge and Planned Nudge.

Participant	No Nudge (%)	Planned Nudge (%)
1	7.41	11.11
2^a^	1.85	0.00
3	26.39	30.09
4	3.24	13.89
5	46.30	52.31
6^a^	3.70	0.00
7	18.06	12.96
8	35.19	38.89
9	1.39	5.56
10	16.67	9.26
11	5.56	7.87
12	6.48	0.93
13	30.09	37.96
14	15.74	42.13
15	20.83	39.35
16	32.87	43.98
17	23.61	37.96
18	38.89	24.54
19	25.93	43.06
20	23.61	16.67

^a^Participant withdrawn.

## Discussion

### Principal Findings

This multiple-period randomized crossover study explored the effect of haptic nudging on UL movement in people with stroke. Haptic nudging increased the likelihood that a person with stroke moved their affected UL by 1.44 times (*P*<.001) during the subacute rehabilitation phase. Haptic nudging resulted in a relative increase in the proportion of affected UL movement of 32.2%. The actual amount and type of UL movement observed without nudging in our study was comparable with our previously published research [16] and other observational studies [33,46]. This strengthens the assertion that haptic nudges influence the amount of affected UL movement in people with stroke. This study is the first to specifically investigate whether haptic nudges delivered by a wrist-worn wearable device influence the amount of UL movement undertaken during rehabilitation following stroke. Given the limited amount of spontaneous UL movement following stroke [16,46,49] and the challenges associated with increasing the dose of UL rehabilitation [15,50], these research findings indicate that haptic nudging represents a potentially powerful stroke rehabilitation tool that could be easily implemented in clinical practice.

Our secondary analysis clarified the type of UL movement promoted by haptic nudging. Participants were 2.03 times more likely to move their affected UL in isolation (unilateral affected UL movement) following a haptic nudge compared with moving the affected UL in concert with the unaffected UL (bimanual movement + bilateral limb movement), which was just 1.13 times more likely. Given that participants were instructed to move their affected UL rather than both limbs together, the instructions related to haptic nudging may be important in determining exactly which movements are promoted. This specificity in effect has been noted in other studies in which people with stroke altered their behavior in response to feedback. Dobkin and Plummer-D'Amato [51] gave daily feedback in relation to gait speed to people with stroke undertaking rehabilitation. They reported the feedback group had significantly increased gait speed but did not change walking endurance or independence compared with the control group (usual care). Our study contributes to the growing body of research suggesting that drawing attention to specific aspects of movement and physical activity throughout the rehabilitation day can influence patient behavior during stroke rehabilitation [26-31].

The greater odds of moving the affected UL in the minute following a planned nudge resulted in a 32.2% increase in the average amount of movement. Another small-scale proof-of-concept study involving people with stroke (n=7) [30] indicated an increase of 19.7% in the average amount of affected UL movement in the hour following a haptic nudge reminder to undertake exercises. In that study, participants were instructed to perform up to 80 repetitions of task-specific training in response to a haptic nudge, but only received a median of 4 nudges across the rehabilitation day. In contrast, participants in our study received an average of 27 haptic nudges and were instructed to move, try to move, or think about moving their affected UL following a nudge. It is not yet known how the frequency of nudges, burden of the required behavioral response, and capacity to integrate that response into everyday activities influence the magnitude and duration of the haptic nudge effect in people with stroke.

Results for individual participants illustrated that there was considerable variability in response to haptic nudging. For example, 13 participants increased the amount of movement of their affected UL in response to nudging, with 8 exhibiting large relative increases. In contrast, 5 participants had a reduction in the amount of affected UL movement in response to haptic nudging. There appeared to be no relationship between individual response and participant age, stroke severity, hemiplegic side, or whether the hemiplegic side was the dominant hand. Although the researchers checked that the participant understood the instruction to move the affected UL in response to haptic nudging at the beginning of data collection, it remains unclear whether cognitive, communication, perceptual, or sensory deficits influenced participants’ ability to attend and respond to the nudge. Other nudging modalities (eg, auditory tones, lights, and text messaging) may be effective and more appropriate than haptic vibration for some people with stroke [52]. In addition, we relied on observation of movement, and it was not possible to determine whether participants who were more severely affected were attempting to or thinking about moving their affected UL. This could be addressed in future research by using alternative data collection methods such as ecological momentary assessment [53] or electroencephalography to determine movement intention [54]. One participant who had moved less in response to haptic nudging had a cerebellar stroke that influenced UL movement bilaterally; advocating increased movement of the more affected limb might have been inappropriate in that case. The relationship between haptic nudge efficacy and clinical and demographic factors requires further investigation to ensure that this type of technology is used appropriately.

The magnitude of effect in response to haptic nudging might have been underestimated in our study given that we included observations in which planned nudges were not delivered (Missed Nudges), for example, when a participant was asleep or not visible to the researcher. This assertion was supported by the exploratory analysis that revealed the OR for the effect of the scheduled nudge when actually delivered compared with no haptic nudge was 1.64. It is also noteworthy that our participants were advised of the value of moving the affected UL after stroke on a single occasion. The effect of haptic nudging may be enhanced by providing regular positively framed information on the consequences of nudged behavior (eg, “more movement promotes recovery”), encouraging explicit action planning (eg, “I will move my affected arm by…when I feel the nudge”), repeated practice of the desired behavioral response to the haptic nudge, and tracking and reporting the desired behavior [35,55,56]. In commercial wearable devices, haptic nudging is commonly coupled with other behavior change and persuasive strategies including education, gamification, social support via social network services, and reward systems [56,57]. It is likely that the magnitude of effect of a comprehensive rehabilitation wearable technology that incorporates haptic nudging with other behavioral change and persuasive strategies would have a larger effect than the effect of haptic nudging alone, as estimated in this study [35,58,59].

This study sought to investigate the effect of haptic nudging on UL movement across an inpatient rehabilitation day; we did not explore the effect on UL movement over a longer timeframe or in a community setting. It is possible that people with stroke habituate or become less responsive to haptic nudging with everyday use. Conversely, they may learn to respond to haptic nudging more effectively over time. Understanding the effect over time is important, as increasing the dose of upper limb movement to a therapeutic level through continued engagement over a matter of weeks to months is likely required to promote functional gains. In the subacute phase following stroke, adherence to wearable devices has been reported as high [31,42], although use appears to dwindle over time [42]. This is consistent with studies in healthy community-dwelling people, where half to two-thirds of purchasers continued to use wearable devices 6 months after purchase [35,36]. In healthy populations, uptake and ongoing use of such devices are influenced by personal characteristics, including age, computer self-efficacy, ease of use, usual levels of physical activity, internalization of intention to change, and personality [36,60]. Although wearable devices have been found to be acceptable to people with stroke [61], one of our participants withdrew at the beginning of data collection because wearing the device made him feel anxious. Previous research indicates that the use of wearable technologies may increase anxiety in clinical populations [62,63]. When developing wearable devices to promote physical activity and movement in people with stroke it may be important to consider the personal and clinical characteristics of the intended users, when and where in the continuum of care the device will be used and for how long, and how users’ engagement and adherence can be supported.

### Limitations

A key limitation of this study was that the researcher observing and recording movement was also responsible for triggering the nudge and therefore not blinded to the intervention. While the randomization schedule was designed to address lag, where the effect of a nudge influences subsequent movement observation periods (refer to Multimedia Appendix 1), the duration of the nudge effect was unknown and might have influenced subsequent observations. While participants were blinded to the study hypothesis, it is possible that the research protocol, particularly being observed by a researcher, may have influenced the likelihood they moved in response to the haptic nudge. Documentation of the number of potential participants screened and the reasons for exclusion from the study would have helped to interpret the external validity of the study findings. A more detailed evaluation of the included participants’ sensorimotor, perceptual, cognitive, and communication impairments along with measurement of their UL functional abilities may have allowed for a more nuanced interpretation of the effect of haptic nudging in people with different clinical presentations of stroke.

### Conclusions

Haptic nudging increased the likelihood that a person with stroke moved their affected UL by 1.44 times. This equated to an increase of 32% in the average amount of affected UL movement. Participants were twice (OR 2.03) as likely to move their affected UL in isolation (unilateral affected UL movement) in response to haptic nudging, compared with movement in conjunction with the unaffected UL (OR 1.13), indicating that the effect of haptic nudging was specific to the behavioral instructions given. Given the limited amount of spontaneous UL movement following stroke and the challenges associated with increasing the dose of UL rehabilitation, haptic nudging as part of a comprehensive wearable device aimed at increasing the dose of UL movement represents a potentially powerful stroke rehabilitation tool.

## Appendix

Multimedia Appendix 1 Statistical analysis for the haptic nudge study.

## Abbreviations

AU: unilateral affected upper limb movement.
AUL: affected upper limb.
BiL: bilateral limb movement.
BiM: bimanual movement.
LACS-H: lacunar circulation syndrome hemorrhagic.
LACS-I: lacunar circulation syndrome ischemic.
OR: odds ratio.
PACS-H: partial anterior circulation syndrome hemorrhagic.
PACS-I: partial anterior circulation syndrome ischemic.
POCS-I: posterior circulation syndrome ischemic.
TACS-I: total anterior circulation syndrome ischemic.
UL: upper limb.
