# Supplementary material for: Haptic Nudges Increase Affected Upper Limb Movement During Inpatient Stroke Rehabilitation: Multiple-Period Randomized Crossover Study
Source: JMIR Mhealth Uhealth. 2020 Jul 29;8(7):e17036. doi: 10.2196/17036 (PMC7424469; doi:10.2196/17036)
Supplement: Multimedia Appendix 1 [file mhealth_v8i7e17036_app1.pdf]

Statistical Analysis for the Haptic Nudge Study

Usman Rashid, Alain C. Vandal

04/11/2019

Contents

1 Randomisation Schedule **2**

2 Variables Included in the Analysis **2**

3 Data Visualisations **3**
3.1 Activity Distribution . . . 3
3.2 Activity Proportions . . . 4
3.3 Reasons for Missed Nudges . . . 5
3.4 Participant-wise Activity Proportions . . . 6
3.5 Participant-wise Proportions for AU, BiL, BiM . . . 7

4 Primary Analysis **8**
4.1 Logistic Mixed Model . . . 8
4.2 Model Estimates with Confidence Intervals . . . 8
4.3 Variance Explained across Time with Natural Splines . . . 9
4.4 Sensitivity Analysis . . . 10
4.4.1 Logistic Mixed Models . . . 11
4.4.2 Sensitivity Estimates with Confidence Intervals . . . 11
4.5 Exploratory Analysis . . . 12
4.5.1 Local Average Treatment Effect (LATE) . . . 12
4.5.2 Logistic Mixed Models . . . 12
4.5.3 LATE Estimate with Confidence Intervals . . . 13
4.5.4 Estimated Compliance across Time . . . 13

5 Secondary Analysis **14**
5.1 Logistic Mixed Models . . . 14
5.2 Model Estimates with Confidence Intervals . . . 15

1 Randomisation Schedule

Haptic nudge reminders were manually triggered by the observer immediately prior to movement observation by the researcher according to a planned randomisation schedule, such that for half of the observation periods a haptic nudge was to be provided and for half a haptic nudge was not to be provided. To account for any potential carry over effect across multiple observation periods randomisation was designed to deliver different types of nudge sequences. These sequences are given in the following table along with their number of occurrences.

Nudge sequence	No. of occurrences
Nudge, No nudge	120, 120
Nudge-Nudge, No nudge-No nudge	120, 120
Nudge-Nudge-Nudge, No nudge-No nudge-No nudge	120, 120
Total	720, 720

2 Variables Included in the Analysis

```
## 'data.frame':    8640 obs. of  7 variables:
##  $ PartID      : Factor w/ 20 levels "1","2","3","4",..: 1 1 1 1 1 1 1 1 1 1 ...
##  $ Time        : Factor w/ 72 levels "1:00 PM","1:10 PM",..: 55 56 57 58 59 60 61 62 63 64 ...
##  $ Obs         : num  1 2 3 4 5 6 7 8 9 10 ...
##  $ Activity    : Factor w/ 6 levels "AU","BiL","BiM",..: 6 5 4 6 5 5 4 4 4 4 ...
##  $ Hour        : Factor w/ 12 levels "1","2","3","4",..: 1 1 1 1 1 1 2 2 2 2 ...
##  $ Reason.DNN  : Factor w/ 7 levels "Inappropriate",..: 3 7 7 3 7 7 7 7 7 7 ...
##  $ Nudge.Status: Factor w/ 4 levels "Nudged","Not nudged",..: 3 2 1 3 1 2 2 2 1 1 ...
```

3 Data Visualisations

3.1 Activity Distribution

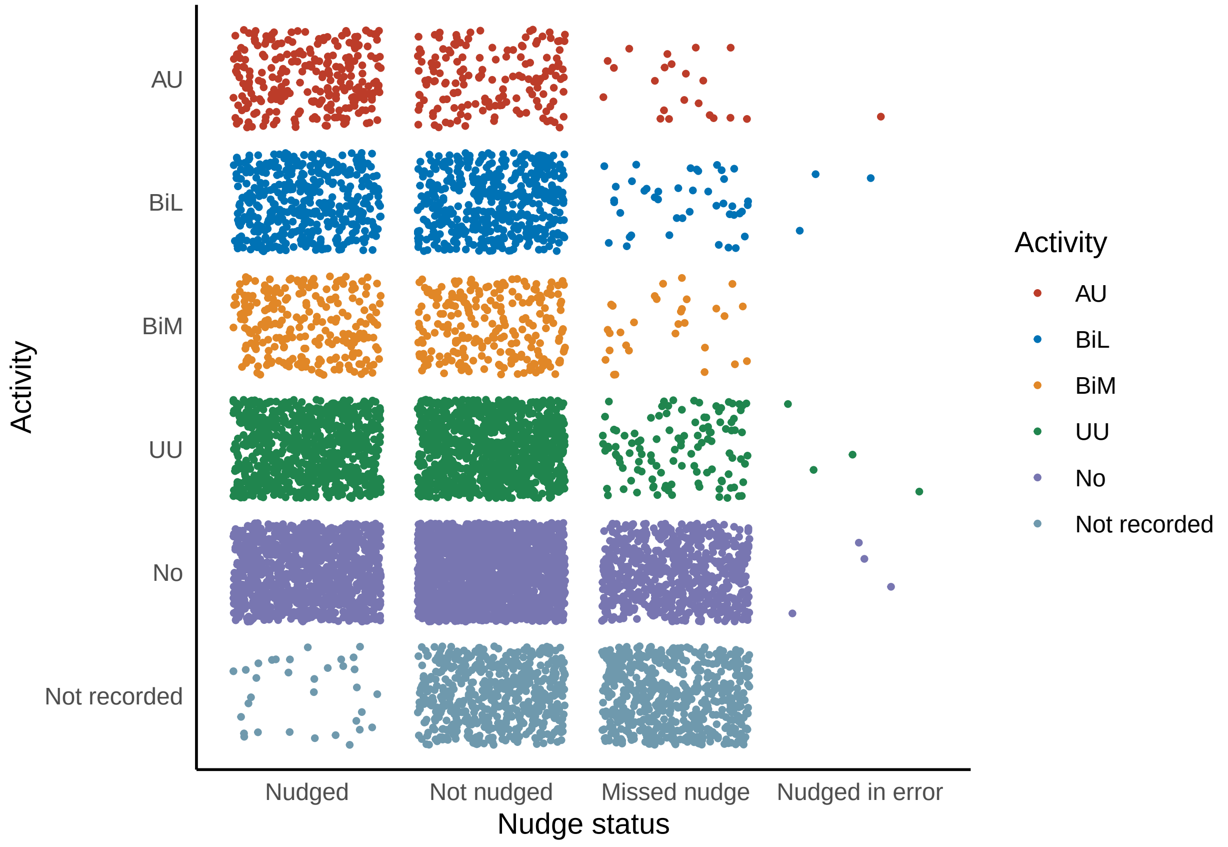

3.2 Activity Proportions

Each planned category (Nudge, No nudge) was to precede half (or 50%) of the total observations. However, it was not always possible to nudge the participants in accordance with the schedule. This resulted in an additional category (Missed nudge). Moreover, in some cases participants were nudged mistakenly when they were not supposed to be nudged. This also resulted in an additional category (Nudged in error).

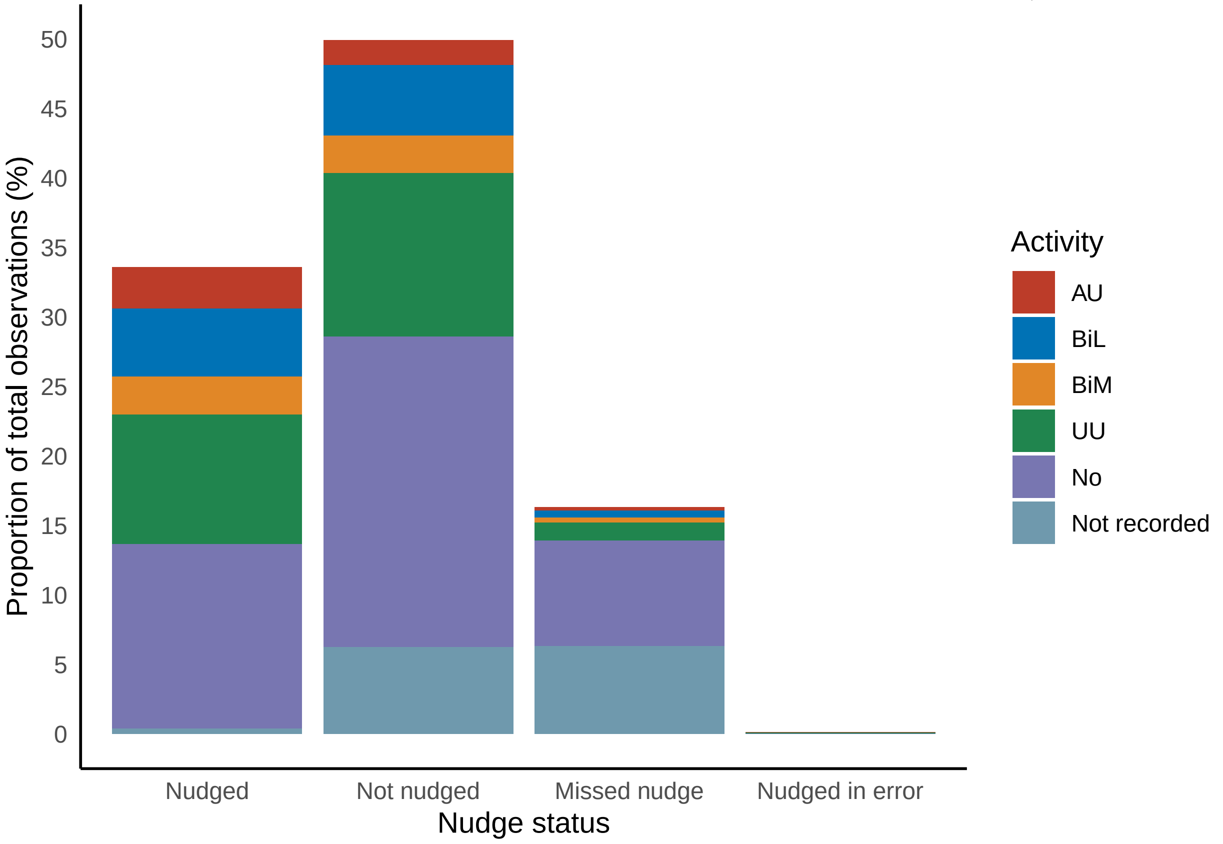

3.3 Reasons for Missed Nudges

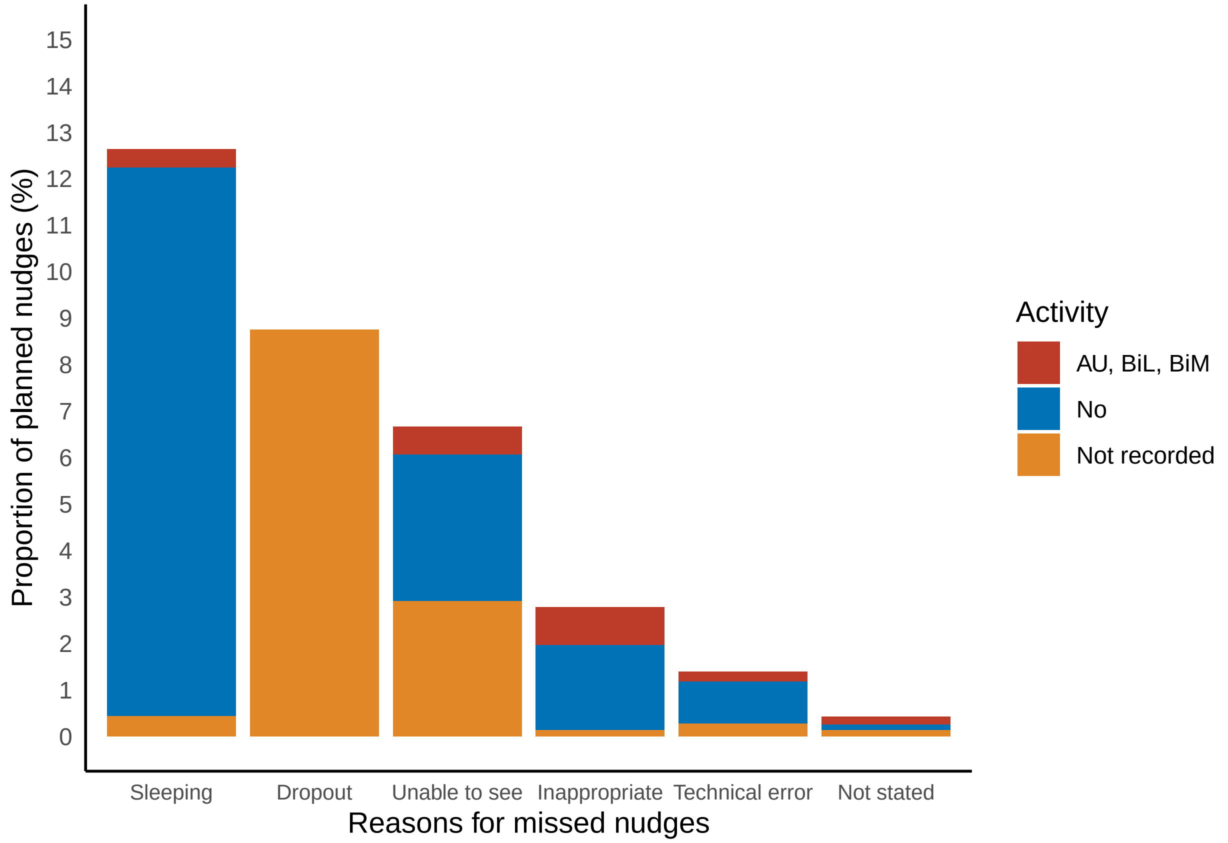

3.4 Participant-wise Activity Proportions

In this and subsequent sections, Nudged and Missed nudge are treated as a single category (Planned nudge), and Not nudged and Nudged in error are both treated as No nudge.

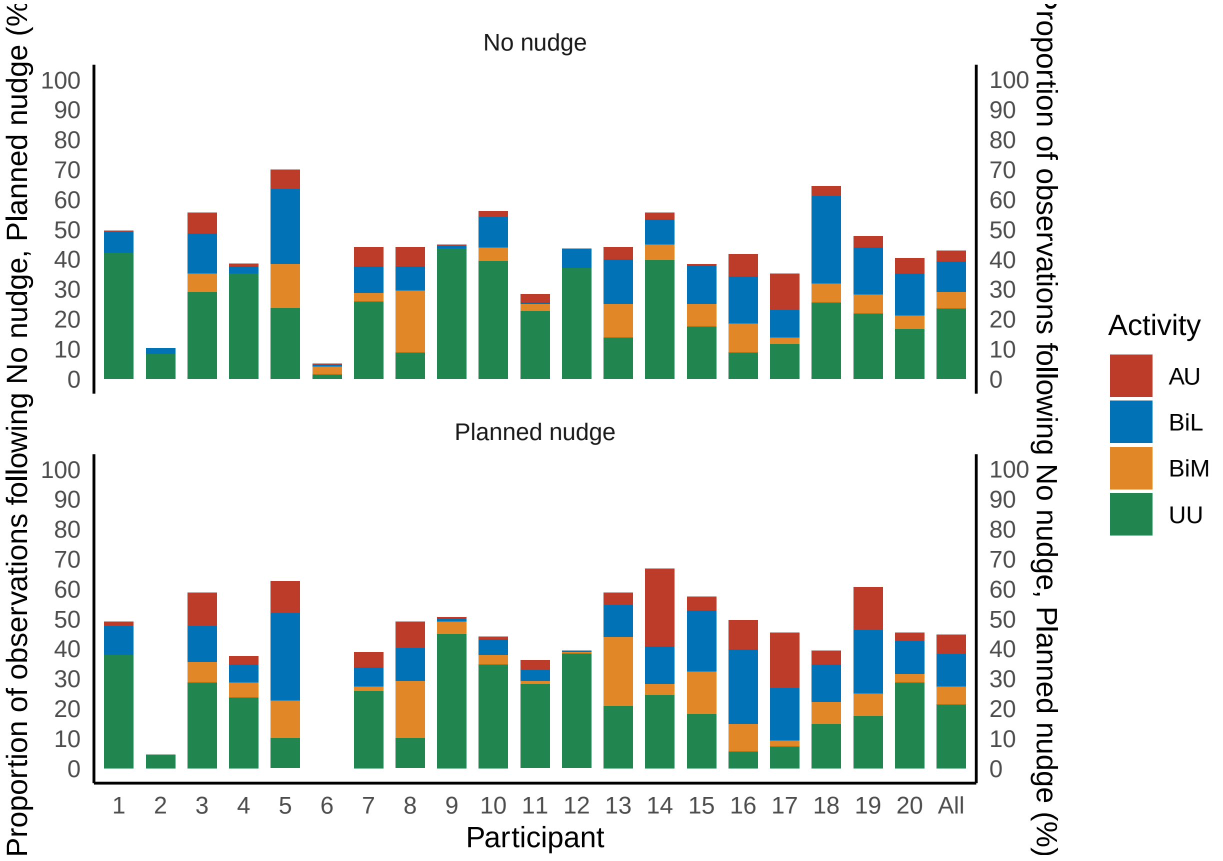

3.5 Participant-wise Proportions for AU, BiL, BiM

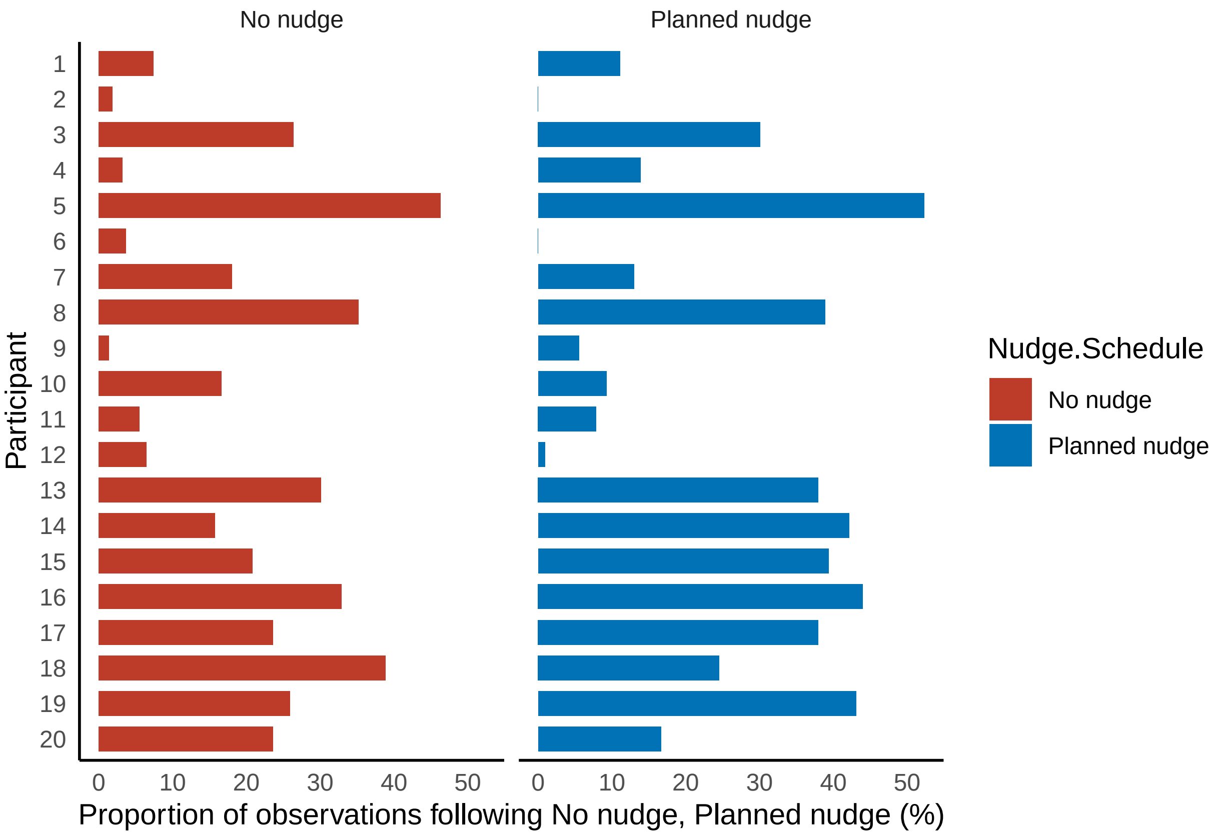

4 Primary Analysis

The aim of this intention to treat analysis is to estimate the effect of Planned nudge compared to No nudge on the combined proportion of AU, BiL or BiM types of activity out of AU, BiL, BiM, UU and No.

```
modelData                 <- dataSource.orig

modelData$Nudge.Schedule  <- factor(modelData$Nudge.Status,
                                    levels = c("Nudged", "Not nudged",
                                               "Missed nudge", "Nudged in error"),
                                    labels = c("Planned nudge", "No nudge",
                                               "Planned nudge", "No nudge"))

modelData$Activity        <- factor(modelData$Activity,
                                    levels = c("AU", "BiL", "BiM", "UU",
                                               "No", "Not recorded"),
                                    labels = c("AU, BiL, BiM", "AU, BiL, BiM",
                                               "AU, BiL, BiM",
                                               "No", "No", "Not recorded"))

levels(modelData$Activity)[levels(modelData$Activity) == "Not recorded"] <- NA

modelData$Activity        <- relevel(modelData$Activity, "No")
```

4.1 Logistic Mixed Model

The model has smooth cubic splines to explain variance across time. The knots are placed at: [8:00 AM, 9:00 AM, 10:00 AM, 11:00 AM, 12:00 PM, 1:00 PM, 2:00 PM, 3:00 PM, 4:00 PM, 5:00 PM, 6:00 PM].

```
lmerModel <- glmer(Activity ~ Nudge.Schedule + ns(Obs, knots = seq(7, 67, 6)) +
                     (1|PartID/Hour),
                   na.action = na.omit,
                   data = modelData,
                   family = binomial(link = "logit"),
                   control = glmerControl(optimizer = "bobyqa"))
```

4.2 Model Estimates with Confidence Intervals

Buzzed	Probability	SE	95% CI lower	95% CI upper
Planned nudge	0.2670266	0.0694465	0.1537880	0.4220589
No nudge	0.2017954	0.0570741	0.1120875	0.3361211

Contrast	Odds ratio	SE	Z-value	P-value	95% CI lower	95% CI upper
Planned nudge / No nudge	1.441018	0.0898571	5.859032	0	1.275238	1.628349

4.3 Variance Explained across Time with Natural Splines

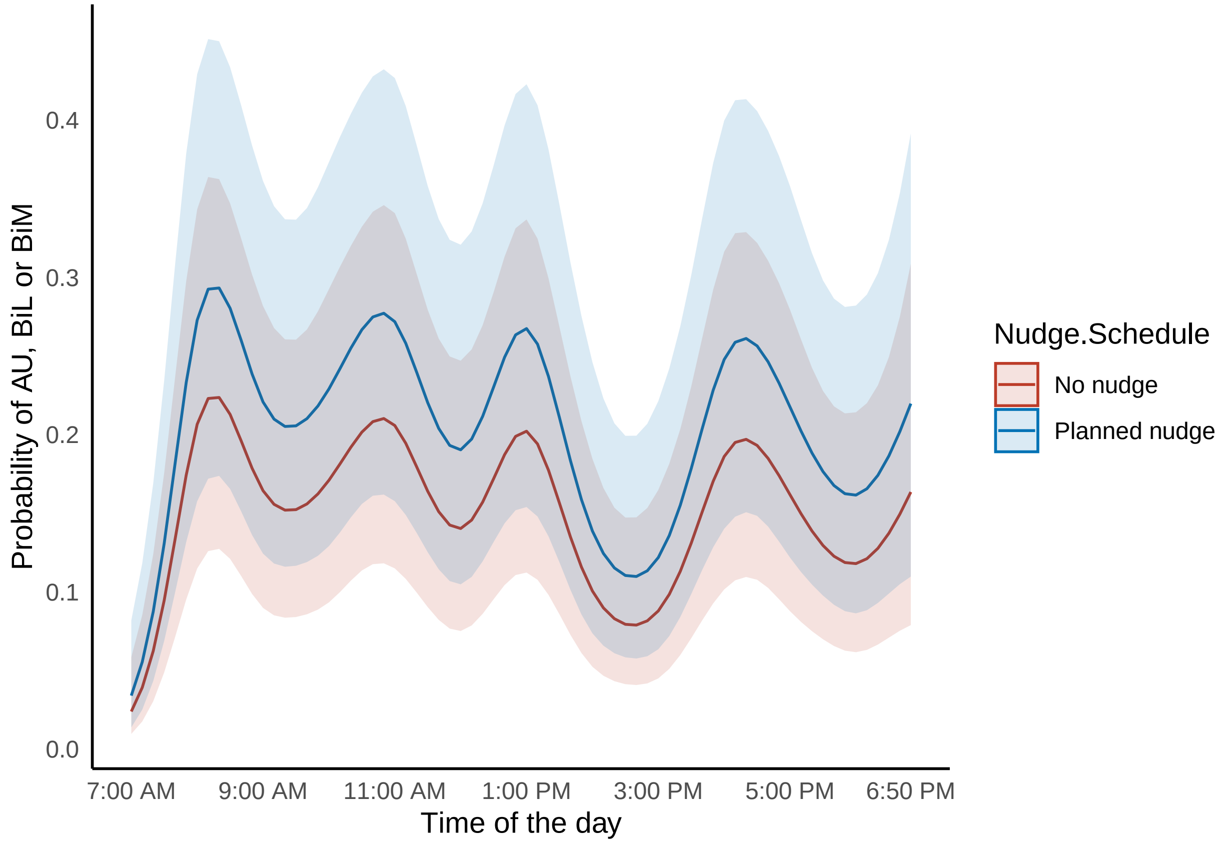

4.4 Sensitivity Analysis

A sensitivity analysis consisting of a simulated worst-case scenario is conducted to evaluate the sensitivity of the estimated effect size for the Planned nudge to missing data. The worst-case simulation is based on multiple (m = 10) random imputations from single trial binomial distributions with mean activity proportions of the participant with worst outcomes.

```
modelData.WC              <- dataSource.orig

modelData.WC$
  Nudge.Schedule          <- factor(modelData.WC$Nudge.Status,
                                    levels = c("Nudged", "Not nudged",
                                               "Missed nudge", "Nudged in error"),
                                    labels = c("Planned nudge", "No nudge",
                                               "Planned nudge", "No nudge"))

modelData.WC$Activity     <- factor(modelData.WC$Activity,
                                    levels = c("AU", "BiL", "BiM", "UU",
                                               "No", "Not recorded"),
                                    labels = c("AU, BiL, BiM", "AU, BiL, BiM",
                                               "AU, BiL, BiM",
                                               "No", "No", "Not recorded"))

levels(modelData.WC$Activity)[levels(modelData.WC$Activity) == "Not recorded"] <- NA

modelData.WC$Activity     <- relevel(modelData.WC$Activity, "No")

list.modelData.WC         <- list()

for(i in seq(1, 10)) { # 10 random imputations

  freshCopy               <- modelData.WC

  # Random imputation
  # Number of missing values following "No nudge"       = 542
  rNN                     <- factor(rbinom(542, 1, 6.4814815/100),
                                    levels = c(0, 1), labels = c("No", "AU, BiL, BiM"))
  # Number of missing values following "Planned nudge"  = 1122 - 542
  rPN                     <- factor(rbinom(1122 - 542, 1, 0.9259259/100),
                                    levels = c(0, 1), labels = c("No", "AU, BiL, BiM"))

  freshCopy$Activity[is.na(freshCopy$Activity) &
                     freshCopy$Nudge.Schedule == "No nudge"]      <- rNN
  freshCopy$Activity[is.na(freshCopy$Activity) &
                     freshCopy$Nudge.Schedule == "Planned nudge"] <- rPN

  list.modelData.WC[[i]]  <- freshCopy
}
```

4.4.1 Logistic Mixed Models

```
list.lmerModel.WC <- list()
for(i in seq(1, 10)) {
  list.lmerModel.WC[[i]] <- glmer(Activity ~ Nudge.Schedule +
                                    ns(Obs, knots = seq(7, 67, 6)) +
                                    (1|PartID/Hour),
                                  na.action = na.omit,
                                  data = list.modelData.WC[[i]],
                                  family = binomial(link = "logit"),
                                  control = glmerControl(optimizer = "bobyqa"))
}
```

4.4.2 Sensitivity Estimates with Confidence Intervals

Case	Nudge.Schedule	Probability	SE	95% CI lower	95% CI upper
WC	Planned nudge	0.1984431	0.0517592	0.1157023	0.3190283
WC	No nudge	0.1599766	0.0437179	0.0914579	0.2648841

Case	Contrast	Odds ratio	SE	Z-value	P-value	95% CI lower	95% CI upper
WC	Planned nudge / No nudge	1.300173	0.0776542	4.394279	1.32e-05	1.156545	1.461639

4.5 Exploratory Analysis

4.5.1 Local Average Treatment Effect (LATE)

To estimate the local average treatment effect (also known as complier average causal effect (CACE)), an instrumental variable analysis is conducted.

```
modelData.IV                <- dataSource.orig

modelData.IV$
  Nudge.Schedule            <- factor(modelData.IV$Nudge.Status,
                                      levels = c("Nudged", "Not nudged",
                                                 "Missed nudge", "Nudged in error"),
                                      labels = c("Planned nudge", "No nudge",
                                                 "Planned nudge", "No nudge"))

modelData.IV$Nudge.Schedule <- relevel(modelData.IV$Nudge.Schedule, "No nudge")

modelData.IV$Nudge.Deliver  <- factor(modelData.IV$Nudge.Status,
                                      levels = c("Nudged", "Not nudged",
                                                 "Missed nudge", "Nudged in error"),
                                      labels = c("Nudged", "Not nudged",
                                                 "Not nudged", "Nudged"))

modelData.IV$Activity       <- factor(modelData.IV$Activity,
                                      levels = c("AU", "BiL", "BiM", "UU", "No",
                                                 "Not recorded"),
                                      labels = c("AU, BiL, BiM", "AU, BiL, BiM",
                                                 "AU, BiL, BiM",
                                                 "No", "No", "Not recorded"))
modelData.IV$Activity       <- relevel(modelData.IV$Activity, "No")

levels(modelData.IV$Activity)[levels(modelData.IV$Activity) == "Not recorded"] <- NA
```

4.5.2 Logistic Mixed Models

```
lmerModel.IV.r        <- glmer(Nudge.Deliver ~ ns(Obs, knots = seq(7, 67, 6))*Nudge.Schedule +
                                 (1|PartID/Hour),
                               family = binomial(link="logit"),
                               modelData.IV,
                               control = glmerControl(optimizer = "bobyqa",
                                                      optCtrl=list(maxfun = 1e6)))

modelData.IV$
  Nudge.Schedule.r    <- resid(lmerModel.IV.r, type = "response")

lmerModel.IV          <- glmer(Activity ~ Nudge.Deliver + Nudge.Schedule.r +
                                 ns(Obs, knots = seq(7, 67, 6)) +
                                 (1|PartID/Hour),
                               na.action = na.omit,
                               data = modelData.IV,
                               family = binomial(link = "logit"),
                               control = glmerControl(optimizer = "bobyqa"))
```

4.5.3 LATE Estimate with Confidence Intervals

Case	Buzzed	Probability	SE	95% CI lower	95% CI upper
LATE	Nudged	0.2857959	0.0724608	0.1663566	0.4451937
LATE	Not nudged	0.1965228	0.0558364	0.1089760	0.3284736

Case	Contrast	Odds ratio	SE	Z-value	P-value	95% CI lower	95% CI upper
LATE	Nudged / Not nudged	1.636041	0.1190303	6.766249	0	1.418616	1.886789

4.5.4 Estimated Compliance across Time

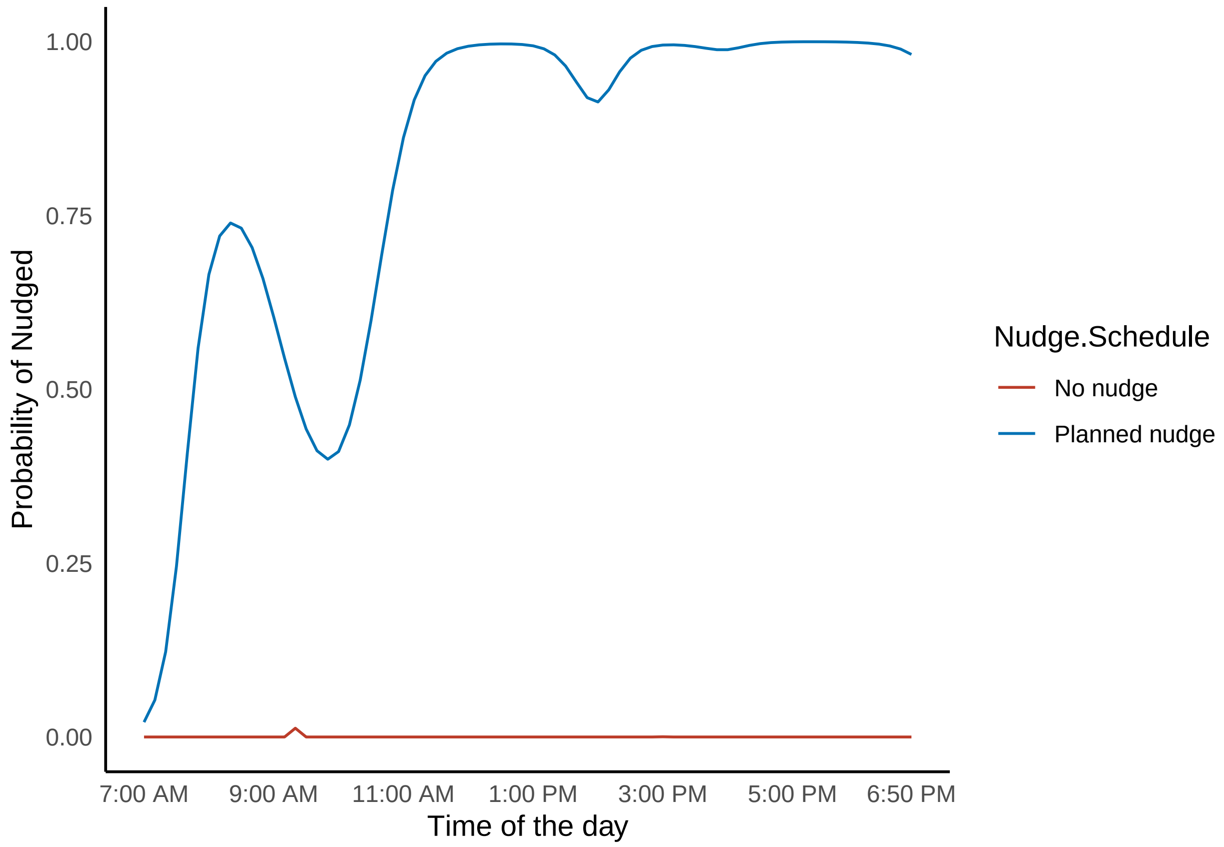

5 Secondary Analysis

The aim of this analysis is to separately estimate the effect of Planned nudge compared to No nudge on the proportion of AU and BiL or BiM types of activity out of AU, BiL, BiM, UU and No.

5.1 Logistic Mixed Models

Two linear logistic mixed regression models are setup to separately estimate the effect of Planned nudge compared to No nudge on AU and BiL or BiM.

```
# Model for AU
modelData_AU              <- dataSource.orig

modelData_AU$
  Nudge.Schedule          <- factor(modelData_AU$Nudge.Status,
                                   levels = c("Nudged", "Not nudged",
                                              "Missed nudge", "Nudged in error"),
                                   labels = c("Planned nudge", "No nudge",
                                              "Planned nudge", "No nudge"))

modelData_AU$Activity     <- factor(modelData_AU$Activity,
                             levels = c("AU", "BiL", "BiM", "UU",
                                        "No", "Not recorded"),
                             labels = c("AU", "No", "No",
                                        "No", "No", "Not recorded"))

levels(modelData_AU$Activity)[levels(modelData_AU$Activity) == "Not recorded"] <- NA

modelData_AU$Activity     <- relevel(modelData_AU$Activity, "No")

lmerModel_AU              <- glmer(Activity ~ Nudge.Schedule +
                                    ns(Obs, knots = seq(7, 67, 6)) +
                                    (1|PartID/Hour),
                                  na.action = na.omit,
                                  data = modelData_AU,
                                  family = binomial(link = "logit"),
                                  control = glmerControl(optimizer = "bobyqa"))

# Model for BiL, BiM
modelData_BiL_BiM         <- dataSource.orig

modelData_BiL_BiM$
  Nudge.Schedule          <- factor(modelData_BiL_BiM$Nudge.Status,
                                   levels = c("Nudged", "Not nudged",
                                              "Missed nudge", "Nudged in error"),
                                   labels = c("Planned nudge", "No nudge",
                                              "Planned nudge", "No nudge"))

modelData_BiL_BiM$
  Activity                <- factor(modelData_BiL_BiM$Activity,
                                   levels = c("AU", "BiL", "BiM", "UU",
                                              "No", "Not recorded"),
                                   labels = c("No", "BiL, BiM", "BiL, BiM",
                                              "No", "No", "Not recorded"))
```

```r
levels(modelData_BiL_BiM$Activity)[levels(modelData_BiL_BiM$Activity) == "Not recorded"] <- NA

modelData_BiL_BiM$
  Activity                <- relevel(modelData_BiL_BiM$Activity, "No")

lmerModel_BiL_BiM         <- glmer(Activity ~ Nudge.Schedule +
                                     ns(Obs, knots = seq(7, 67, 6)) +
                                     (1|PartID/Hour),
                                   na.action = na.omit,
                                   data = modelData_BiL_BiM,
                                   family = binomial(link = "logit"),
                                   control = glmerControl(optimizer = "bobyqa"))
```

5.2 Model Estimates with Confidence Intervals

Contrast	Odds ratio	SE	Z-value	P-value
Planned nudge / No nudge (AU)	2.034658	0.2172070	6.65390	0.0000000
Planned nudge / No nudge (BiL or BiM)	1.128862	0.0760345	1.79957	0.0719286

Contrast	95% CI lower	95% CI upper
Planned nudge / No nudge (AU)	1.6505268	2.508189
Planned nudge / No nudge (BiL or BiM)	0.9892548	1.288171
